# Problématique de la prise en charge des cancers du sein au Sénégal: une approche transversale

**DOI:** 10.11604/pamj.2016.25.3.3785

**Published:** 2016-09-06

**Authors:** Serigne Modou Kane Gueye, Mamour Gueye, Sophie Aminata Coulbary, Alassane Diouf, Jean Charles Moreau

**Affiliations:** 1 Clinique Gynécologique et Obstétricale CHU A, Le Dantec de Dakar, Sénégal; 2 Centre Hospitalier National de Pikine, Pikine, Sénégal

**Keywords:** Cancer du sein, diagnostic, traitement, Sénégal, Breast cancer, diagnosis, treatment, Senegal

## Abstract

L’heure où les thérapeutiques innovantes se multiplient dans le cancer du sein, des pays moins nantis comme le Sénégal accusent encore un retard considérable dans la prise en charge globale de ce type de cancer. Au Sénégal, même si la prise en charge des cancers du sein avancés est actuellement bien codifiée, les résultats en terme de survie et de morbidités sont encore médiocres vu les retards diagnostiques et les traitements mutilants, parfois onéreux et mal tolérés, devenus nécessaires. Pour ces cancers avancés, les défis qui restent à relever résident dans l’érection de centres de soins palliatifs et le développement de la pluridisciplinarité pour améliorer la qualité de vie et l’accompagnement des malades. En revanche, pour les cancers infracliniques ou potentiellement guérissables, les défis restent immenses car il s’agira de les dépister, de bien les localiser et les diagnostiquer aussitôt (biopsie écho guidée ou stéréotaxique) mais également de les opérer de façon précise et entière (repérage – exérèse in sano et radiographie de pièce opératoire) tout en limitant les complications comme celles du curage classique (biopsie du ganglion sentinelle). Il s’agit là autant d’objectifs auxquels nos structures de santé ne sont pas toujours préparées. Cette mise au point est une analyse situationnelle sur les écueils contextuels qui grèvent encore la prise en charge globale des cancers du sein au Sénégal.

## Abstract (en)

At a time when innovative therapies in breast cancer multiply, poorer countries such as Senegal are still lag far behind in the overall management of this type of cancer. In Senegal, although the treatment of advanced breast cancer is now well codified, survival and morbidity outcomes are still mediocre in view of diagnostic delays and of sometimes expensive and poorly tolerated mutilating treatments become necessary. With respect to advanced cancers, the challenges will lie in building of palliative care centres and in developing multidisciplinary approaches to improve quality of life and to support patients. On the other hand, with respect to preclinical or potentially curable cancers, the challenges are immense given the importance of early detection, localisation and diagnosis (stereotactic or ultrasound guided biopsy) but also of precision surgery and of complete resection (indexing - excision ensuring a margin of healthy tissue and specimen radiograph) while minimizing complications such as those of classic dissection (sentinel lymph node biopsy). Our health structures are not always prepared to achieve these goals. This is a situational analysis of the contextual obstacles that still exist and add a burden on the overall management of breast cancer in Senegal.

## Editorial

Estimé à plus d’un million de cas par an, le cancer du sein est le premier cancer de la femme dans le monde. Aussi, il fait partie des cancers les plus graves avec une mortalité non négligeable; sur 100 femmes qui meurent d’un cancer, 25 sont atteintes de cancer du sein. Dans de nombreux pays développés, réduire la mortalité liée à ce cancer est un défi majeur de santé publique [1]. Dans la plupart des pays en développement, ce cancer est placé au deuxième rang des cancers de la femme après celui du col avec une incidence et une mortalité, sans cesse croissantes [2]. Dans ces pays comme au Sénégal, il persiste encore d’innombrables obstacles à la prise en charge de ce cancer. Au travers de cette revue nous nous proposons de faire une analyse situationnelle des écueils épidémiologiques, diagnostiques et thérapeutiques eu égard aux référentiels de prise en charge actuels de la maladie. Nous résumons cette analyse autour de quelques facteurs compromettants de la prise des cancers du sein au Sénégal.

### Préoccupations épidémiologiques

L’incidence du cancer du sein varie selon de nombreux critères dont la région géographique, l’origine ethnique, le mode de vie et les catégories socio-économiques. Actuellement, on peut estimer son incidence à plus d’un million de cas par an à l’échelle mondiale [1]. Si dans les pays occidentaux l’incidence de ce cancer accuse des valeurs élevées, elle est parfois dix fois plus faible dans certaines régions de Chine. Il fait partie des cancers les plus graves avec une mortalité non négligeable ; sur 100 femmes qui meurent d’un cancer, 25 sont atteintes du cancer du sein. Dans la plupart des pays d’Afrique subsaharienne comme le Sénégal il manque encore de données statistiques fiables à l’échelle nationale voire sous régionale du fait de l’absence de registre des cancers. Ces insuffisances statistiques aussi bien sur l’incidence que sur la mortalité liée à ce cancer masquent l’ampleur du problème et détournent les stratégies vers d’autres priorités non moins préoccupantes (le paludisme, la tuberculose, le VIH/SIDA, l’insuffisance rénale, etc.). Cette problématique statistique affecte également l’épidémiologie analytique avec des questions sans réponses autour d’éventuels facteurs de risques spécifiques ou contextuels propres à la catégorie socio-économique et démographique de la femme sénégalaise. Dans la littérature européenne et occidentale, on décrit clairement les facteurs de risque jusque là bien connus (l’âge, les mutations génétiques délétères, l’hyperoestrogènie endogène ou exogène, certaines mastopathies). Toutefois, au Sénégal comme dans la majorité des pays africains subsahariens, les auteurs décrivent un profil de risque différent chez les patientes atteintes de cancer du sein [3]. En effet, dans cette population: l’âge de survenue du cancer est plus jeune variant entre 35 et 45 ans [4, 5]; l’âge moyen des ménarches est plus tardif estimé à 15 ans [4]; l’âge au premier enfant est fréquemment inférieur à 30 ans [5]; l’allaitement maternel est largement pratiqué entre 69,9% et 90% [4, 5]; l’utilisation de la contraception hormonale est faible, 22% au Sénégal [4]; peu de femmes ménopausées (3,3%) ont recours au traitement hormonal substitutif [3]. Ce profil suscite des interrogations sérieuses autour des véritables facteurs de risque d’un cancer dont l’incidence ne cesse de croitre. En l’absence d’une maitrise parfaite de ces facteurs de risque toute tentative de sensibilisation ou de prévention primaire à l’endroit des populations peut s’avérer plus difficile.

### Limites dans le dépistage

Dans la plupart des pays du Nord, la stratégie de prévention secondaire ou de dépistage des cancers du sein est bien codifiée. Au Sénégal comme dans certains pays en développement, il demeure encore des écarts considérables dans l’application effective des méthodes de dépistage.

### L’auto-examen des seins (AES)

**Figure 1:**
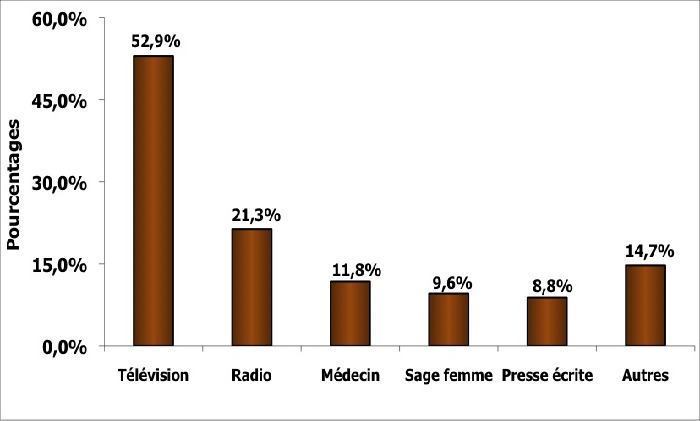
Sources de l’information sur l’AES au Sénégal

Il est faiblement pratiqué au Sénégal; 29% selon une enquête hospitalière [3] du fait d’un faible niveau de connaissance sur l’AES, mais surtout d’une rare implication (21,4%) du personnel de santé dans la sensibilisation des populations (Figure 1). Selon plusieurs auteurs, le message de sensibilisation serait mieux transmis par les professionnels de la santé et que, dans ce cas, il aboutirait à une pratique plus régulière de l’AES [6]. Il serait alors intéressant d’orienter la formation initiale et continue du personnel de santé, et particulièrement des conseillers, assistants sociaux et relais communautaires vers l’enseignement de l’AES au profit de la population féminine. Aussi, il faut encourager l’utilisation des médias, l’implication des leaders d’opinion, des artistes et des collectivités pour booster la sensibilisation de proximité au profit des populations moins accessibles.

**L’examen clinique des seins**: la réalité sénégalaise est que l’examen clinique des seins est une pratique exclusivement réservée aux gynécologues, cancérologues et sages femmes. Les taux de réalisation sont globalement très faibles (entre 5 et 26%) aussi bien dans la pratique africaine qu’européenne ou occidental [7]. Dans un contexte de sous équipement nous gagnerons à impliquer davantage le personnel de santé dans l’effort de dépistage du cancer du sein, en intégrant l’examen systématique des seins dans l’examen général de toute patiente.

**La mammographie**: Cet examen constitue le meilleur outil de dépistage à grande échelle. Dans les pays comme la France le dépistage de masse est organisé pour les femmes entre 50 à 74 ans, selon les règles suivantes: mammographie bilatérale, deux incidences, réalisées tous les deux ans avec double lecture. Dans notre contexte où le cancer concerne en majorité une population plus jeune, un dépistage mammographique à partir de 40 ans serait tout à fait justifié. Cependant la mammographie est encore inaccessible à la majorité de la population sénégalaise du fait d’une mauvaise répartition territoriale des centres d’imagerie qui en disposent ; l’essentiel des sénographes n’étant disponible qu’à Dakar et surtout dans les structures privées. En outre, le coût de cet examen est élevé, entre 40000 FCFA (60 Euros) et 80000 FCFA (120 Euros), largement au dessus des possibilités financières du sénégalais moyen. Il faut aussi déplorer la qualité de certains clichés qui ne répondent pas souvent aux critères de validités ou d’interprétation requis pour servir de test de dépistage. De gros efforts de formation, de décentralisation et d’exonération méritent d’être déployés dans ce domaine de la radiologie pour vulgariser la mammographie aux fins de dépistage.

**Le dépistage des cas particuliers**: Il s’adresse aux patientes jeunes avec des seins denses, présentant un haut risque de cancers du sein (antécédents personnels, antécédents familiaux, mutations délétères de type BRCA1 ou BRCA2, certaines mastopathies atypiques, etc.). La mammographie numérique prend tout son intérêt en cas de seins denses. Pour le groupe à haut risque, la stratégie de dépistage recommandée procède par un examen clinique des seins tous les 6 mois, une échographie et une IRM mammaire réalisées tous les ans. Devant des antécédents de cancer du sein dans la famille proche, ce dépistage particulier devra être démarré à partir de 25 à 30 ans ou 5 ans avant l’âge de survenue du premier cancer du sein dans la famille. Au Sénégal, cette forme de dépistage n’est prescrite que par quelques prestataires plus avertis au profit d’une minorité féminine qui peut se payer aussi régulièrement ces examens. Ainsi, en pays pauvre, les stratégies de prévention (secondaire) devraient surtout porter l’accent sur la sensibilisation, les méthodes de dépistage à moindre coût comme l’auto examen des seins et l’examen clinique des seins en attendant la vulgarisation et l’accessibilité financière de la mammographie.

### Problématique du diagnostic

Sans nul doute le diagnostic de certitude du cancer du sein relève de l’histologie. Dans notre contexte, la majorité des patientes (75%) consultent tardivement, à des stades avancés de la maladie [4] où le cancer est palpable et visible à l’œil nu sous forme de volumineuse masse, d’ulcération ou d’inflammation généralisée des seins. A ces stades, le diagnostic est très souvent facile et ne requiert qu’une confirmation par une biopsie au tru-cut. Toutefois, les efforts de sensibilisation et les éventuels succès de dépistage vont nous amener à découvrir des lésions infracliniques, de plus en plus petites et donc non palpables. Dans ce cas, il sera alors nécessaire de recourir à la mammographie et à l’échographie, parfois même à des examens morphologiques plus affinés comme l’IRM pour approcher le diagnostic lésionnel et topographique des « petits » cancers du sein. Ces examens devront aussi aider à orienter et à réaliser des biopsies en profondeur grâce à un repérage échographique ou mieux stéréotaxique. Ces formes de biopsie ne sont pas encore de pratique courante au Sénégal. Actuellement au Sénégal nous faisons beaucoup recours à la cytologie (cytoponction) pour poser le diagnostic de la majorité des pathologies mammaires. Elle présente des avantages (facile à réaliser, rapide, indolore, cellules vivantes et fraîches, diagnostic immédiat, coût faible, idéale pour les kystes avec, dans ce cas, un double rôle diagnostique et évacuateur). Cependant la cytologie nécessite un anatomopathologiste expérimenté et présente quand même des limites. Elle n’a de valeur que positive, le matériel peut être absent ou insuffisant (dépendant du préleveur), elle ne renseigne ni sur le diagnostic d’infiltration ni sur les marqueurs histopronostiques et elle peut jouer des pièges sur les atypies. Les atypies en cytologie requièrent beaucoup d’attention car on peut observer des figures d’atypies cytomorpholgiques au sein du tissu normal à certaines périodes de la vie génitale comme la péripuberté, la grossesse, la périménopause et la ménopause [8]. Ceci rend difficile l’interprétation des échantillons prélevés par aspiration fine. Alors, avant de traiter sur la base d’atypies évoquées à la cytologie, il serait important de confirmer la lésion par une biopsie. Ce reflexe de la biopsie facile devait être développée davantage du fait de ses multiples avantages. Elle permet une analyse épithéliale complète, un diagnostic certain d’invasion, elle accède aux petites lésions non palpables (micro ou macro biopsie stéréotaxique ou échoguidée) et, pour une meilleure évaluation histopronostique, elle permet de faire les marqueurs les plus usuels (RE, RP, HER 2, Ki 67, etc.). Ces renseignements permettent surtout de mieux connaître la nature histologique du cancer et optimiser le traitement. A l’heure actuelle, au Sénégal, il faudrait s’équiper et ériger des unités fonctionnelles pour améliorer le diagnostic des lésions infracliniques car ce sera la réponse pratique à apporter face à une stratégie de sensibilisation et de dépistage qui aura bien fonctionné.

### Limites dans la chirurgie

Le chirurgien reste un acteur principal dans le traitement des tumeurs solides. De la qualité du contrôle locorégional qu’il est capable d’assurer dépend en grande partie le pronostic ultérieur. Comme l’on peut s’y attendre dans notre contexte avec les retards diagnostiques observés (plus de 75% de cancers avancés), l’essentiel de la chirurgie du sein se résume très souvent en un geste très mutilant comme la mastectomie. C’est à dire l’exérèse monobloc de la glande en conservant les deux muscles pectoraux et réalisant un curage axillaire des deux premiers étages de Berg (mastectomie radicale modifiée). Elle ne pose aucune difficulté pratique sauf pour les cancers inflammatoires, exulcérés, avec d’énormes pertes cutanées pouvant poser un problème de fermeture cutanée. Là où nous devons améliorer notre pratique c’est dans le traitement conservateur, qui depuis les années 1970, est devenu le traitement de référence [9] pour les tumeurs uniques de moins de 2 cm. Ici l’exérèse est réalisée au large de la tumeur en préservant un résultat esthétique correct. L’exérèse des lésions non palpables (zonectomie) nécessite un repérage radiologique préopératoire : mise en place d’un harpon ou d’un hameçon sous échographie, mammographie ou IRM ; repérage échographie avec marqueur cutané ; repérage orthogonale où le chirurgien s’oriente par rapport aux clichés de mammographie (face et profil) avec repérage du mamelon par une bille radio-opaque. Cette exérèse radio-chirurgicale nécessite une radiographie de pièce opératoire et parfois une chirurgie de rattrapage (en cas de marges non saines) qui reste limitée par le coût des ré-interventions entièrement à la charge des patientes ce qui n’encourage pas le discours d’une chirurgie conservatrice. Le traitement conservateur des lésions palpables (tumorectomie) ne pose pas de problème pratique dans notre contexte. Néanmoins il faut promouvoir la chirurgie oncoplastique pour en garantir l’efficience cosmétique. La transition de la mastectomie vers les traitements conservateurs du sein affecte également le curage axillaire qui doit évoluer vers la biopsie du ganglion sentinelle pour améliorer la qualité de vie des patients et réduire la morbidité liée au curage classique. A l’heure actuelle, ces méthodes de détection et d’exérèse radio-chirurgicale des lésions non palpables et des ganglions sentinelles axillaires ne sont pas encore disponibles au Sénégal ; ce qui limite la qualité de la prise en charge chirurgicale des lésions infracliniques. Dans nos pays, d’importants efforts d’investissement et d’équipement sont nécessaires pour permettre au chirurgien de jouer ce rôle premier et capital dans le traitement des cancers du sein infracliniques.

### Problématique des traitements complémentaires

A l’heure actuelle on reste encore confronté à la découverte tardive (75%) des cancers du sein. Cette situation expose la patiente à un traitement plus mutilant, plus long, plus couteux, moins supportable avec des chances de guérison plus faibles. En plus de la chirurgie, il faudra souvent recourir à la chimiothérapie, à la radiothérapie, à l’hormonothérapie, à l’immunothérapie, aux traitements palliatifs, à la psychothérapie et aux rigueurs de la surveillance. Tel est le parcours habituel des patientes atteintes de cancer du sein en pays pauvres ou intermédiaires. La chimiothérapie est pratiquée avec des protocoles allant des moins couteux (CMF) aux plus onéreux (incluant les taxanes) avec des coûts pouvant aller de 60000 FCFA (100 Euros) à plus de 400000 FCFA (600 Euros) par cure ; compte non tenu des coûts indirects. En dehors des coûts exorbitants de ces produits, leur disponibilité dans le marché pose également problème ; les ruptures sont fréquentes obligeant à des interruptions ou des reports de cures. Cette irrégularité de la chimiothérapie est connue comme facteur de croissance cellulaire tumorale ou d’échappement thérapeutique. Cette difficulté d’accès au traitement se pose également avec les drogues de l’immunothérapie et de la thérapie ciblée (trastzumab, bevacizumab) dont le coût dépasse très souvent et largement les moyens de la population concernée. A l’heure actuelle, la radiothérapie au cobalt est pratiquée au Sénégal par le seul centre anticancéreux existant à Dakar posant le problème de la surcharge de cette unité, augmentant ainsi les délais du traitement avec toujours un coût non négligeable (150000 FCFA soit environ 200 Euros). Au Sénégal, comme ailleurs dans le monde, la radiothérapie pariétale après mastectomie et celle des aires ganglionnaires répondent aux mêmes indications et exigences techniques. L’irradiation de l’ensemble de la glande dans le cadre du traitement conservateur du sein reste également le traitement de référence [10]. Cependant, les contraintes économiques, les retards thérapeutiques et la surcharge du seul centre de Dakar doivent amener à s’orienter vers d’éventuelles innovations thérapeutiques, basées sur des concepts comme l’irradiation partielle du sein (IPS) ou l’irradiation partielle accélérée du sein (IPAS). Ces techniques permettraient de réduire le risque des « vraies » récidives tout en raccourcissant les délais du traitement mais elles doivent être validées par des études rigoureuses. Le tamoxifène reste le traitement antihormonal le plus utilisé dans les cancers exprimant les récepteurs hormonaux [10]. Son coût abordable (3000 FCFA soit 5 Euros par mois en générique ou 16000 FCFA soit 25 Euros par mois en spécilaité) constitue, entre autres, un critère de sa large prescription d’autant que la durée du traitement est en moyenne de 5 ans. Chez les femmes ménopausées ou présentant un risque thrombo-embolique ou de cancer de l’endomètre, les anti-aromatases sont largement indiqués, mais ils sont encore onéreux au Sénégal (120 Euros par mois). Ces difficultés d’accès aux traitements indiqués exposent souvent à la progression de la maladie vers les stades de cancers localement avancés, métastatiques, dans des formes parfois monstrueuses, confinant le patient dans le désespoir et lui réservant que des soins palliatifs dans un pays où il n’existe pas de centres dédiés à ce type de soins. L’objectif encore manqué est celui de l’accompagnement des patientes et la précocité des traitements ; ce qui, naturellement, nous éloigne des référentiels de prise en charge du cancer du sein.

### Besoins en soins palliatifs

Dans un contexte où les cancers sont découverts, en majorité, à des stades tardifs, les soins palliatifs trouvent toute leur importance. Ils regroupent tous les soins locaux des cancers infectés, non opérables, les soins face aux complications de la chirurgie, de la radiothérapie ou de la chimiothérapie, les altérations de l’état général et les complications propres à la maladie évolutive. A ces stades où les moyens habituels sont « dépassés » avec peu de chances de guérison, l’accompagnement de la patiente par les soins locaux, la rééquilibration métabolique, la réanimation, la nutrition, la psychothérapie sont autant de méthodes capitales et utiles dans l’amélioration de la qualité de vie des patientes. Dans notre contexte, on doit promouvoir des centres de soins palliatifs qui concentreraient l’essentiel de ces méthodes pluridisciplinaires pour répondre à une demande de soins de plus en plus croissante et témoin des écueils considérables dans la prise en charge des cancers du sein au Sénégal.

### Contribution de l’Etat et des partenaires

La prise en charge des cancers du sein est une tache multisectorielle et nécessite une volonté politique devenue urgente au Sénégal. Lutter contre le cancer dans un contexte de pays intermédiaire va nécessiter une interposition de l’état et des partenaires entre un patient de plus en plus pauvre (du diagnostic au traitement) et des thérapeutiques de plus en plus affinées et donc de plus en plus onéreuses. L’autorité sanitaire doit promouvoir l’organisation du registre des cancers afin de mieux appréhender l’immensité du phénomène, appuyer les programmes de lutte contre le cancer en rendant accessibles les outils de dépistage de masse, encourager la formation de personnels compétents dans le domaine. Néanmoins, tout ne saurait être réalisé par l’état. C’est l’occasion de louer le travail considérable mené par la Ligue Sénégalaise de Lutte contre le Cancer (LISCA), les travailleurs sociaux, les partenaires et les collectivités dans le soutien aux patients démunis. Il faut enfin inviter tous les partenaires et acteurs de la santé, locaux comme étrangers, à s’impliquer davantage dans la lutte contre les maladies émergentes comme le cancer du sein ; entre autres.

Cette analyse situationnelle nous a permis d’identifier les écueils persistants dans la prise en charge des cancers du sein au Sénégal. Elle nous rappelle les grands écarts à combler dans la sensibilisation des populations mais également dans la formation et l’équipement des structures afin de pouvoir améliorer le diagnostic, conduire une prise en charge proche des référentiels à jour et enfin améliorer l’accompagnement psycho-social des patients. L’atteinte de ces objectifs passent nécessairement par l’amélioration de la disponibilité et de l’accessibilité des médicaments anticancéreux et le développement d’unités fonctionnelles compétentes dans le diagnostic, la chirurgie mammaire, les soins palliatifs dans un environnement qui promeut la concertation pluridisciplinaire.
